# Poor treatment outcomes of acute exacerbations of chronic obstructive pulmonary disease and their associated factors among admitted patients in East Gojjam, 2023

**DOI:** 10.3389/fmed.2024.1434166

**Published:** 2024-11-20

**Authors:** Melaku Tadele Getnet, Abebe Dilie Afenigus, Menberu Gete, Ambaw Abebaw Emrie, Dejen Tsegaye

**Affiliations:** ^1^Dejen Primary Hospital, Gojjam, Ethiopia; ^2^Department of Nursing, College of Health Sciences, Debre Markos University, Debre Markos, Ethiopia; ^3^Department of Pediatrics and Child Health, College of Health Sciences, Wolkite University, Wolkite, Ethiopia

**Keywords:** acute exacerbation, chronic obstructive pulmonary disease, admitted patients, Debre Markos comprehensive referral hospital, poor treatment outcome

## Abstract

**Background:**

Acute exacerbation of chronic obstructive pulmonary disease (COPD) poses a significant public health challenge globally, resulting in considerable health and economic burden. To date, there has been insufficient research in Ethiopia regarding poor treatment outcomes associated with these acute exacerbations.

**Objective:**

This study aims to assess the poor treatment outcomes of acute exacerbations of chronic obstructive pulmonary disease and identify the associated factors among admitted patients in East Gojjam in 2023.

**Design:**

An institutional-based cross-sectional study design was employed.

**Methods:**

The institutional-based cross-sectional study was conducted from 7 April 2023 to 7 May 2023, involving 384 participants selected through simple random sampling. Data were extracted from patient charts and registers. Data entry was performed using EpiData, and the analysis was conducted using IBM SPSS Statistics version 26 software. Binary logistic regression analysis was used to identify the association between dependent and independent variables. Variables with a *p*-value of <0.25 in the bivariable logistic regression analysis were considered candidates for multivariable logistic regression. Variables with a *p*-value of <0.05 were considered statistically significant.

**Results:**

Out of a total of 346 patients, 99 (28.6%) (95% CI, 23.9–33.3) developed poor treatment outcomes following exacerbations of chronic obstructive pulmonary diseases. Poor treatment outcomes were significantly associated with the following variables: age 65 or older (AOR = 3.9; 95% CI: 1.57–9.71), presence of comorbidities (AOR = 2.6; 95% CI: 1.287–5.20), a hospital stay longer than 7 days (AOR = 3.9; 95% CI: 1.97–7.70), and low oxygen saturation (<88%) (AOR = 9.0; 95% CI: 4.43–18.34).

**Conclusion:**

Approximately one-third of the patients treated for acute exacerbations of chronic obstructive pulmonary disease at the Debre Markos Comprehensive Specialized Hospital experienced poor treatment outcomes. There is a significant association between poor treatment outcomes of acute exacerbation of chronic obstructive pulmonary disease and age ≥ 65 years, having comorbidities, prolonged hospital stay, and low oxygen saturation.

## Introduction

Acute exacerbations of chronic obstructive pulmonary disease (COPD) refer to the sudden worsening of respiratory symptoms from a stable state that may require treatment and/or hospitalization (1, 2).

Patients experiencing exacerbations of COPD often have systemic comorbidities that can lead to unscheduled hospitalizations and a deterioration of physical health, resulting in a poor prognosis (3). The outcomes of acute exacerbations can include worsening symptoms, increased mortality, complications, and changes in lung function (4, 5). Evidence shows that while some patients with exacerbations of COPD improve and are discharged from the hospital, others experience poor outcomes and develop complications (6).

COPD is a progressive and life-threatening airway disease characterized by breathlessness and a predisposition to exacerbations and serious illness. It is an irreversible condition associated with progressive airflow limitation, manifesting symptoms such as cough, dyspnea, and sputum production (7).

Although acute exacerbations of COPD can be treated, the underlying disease cannot be cured once developed. People with exacerbations of COPD must exert more effort to breathe, which can lead to shortness of breath and fatigue. As the illness progresses, it may become increasingly difficult to exhale or inhale (8, 9).

Exacerbations of COPD are a major public health issue, incurring substantial healthcare and economic costs. Globally, the condition affects over 200 million people and is the fourth leading cause of death, contributing to chronic diffuse irreversible airflow obstruction, mainly in the small airways (10). Projections indicate that by 2030, COPD exacerbations could become the third leading cause of death (11, 12).

Data from the World Health Organization (WHO) reveal that outcomes for patients with exacerbations of COPD are significantly worse in developing countries. Over 90% of related deaths occur due to inadequate access to treatment and a lack of COPD prevention and management strategies (13). Findings from the Global Burden of Disease survey indicated that in 2019, there were 212.3 million prevalent cases of COPD with 3.3 million deaths globally (14). COPD is also a leading cause of hospital admissions, reduced quality of life, poor outcomes, and increased healthcare costs. Unscheduled medical visits and increased healthcare resource utilization are primarily driven by acute exacerbations of COPD (15–17).

The socioeconomic burden of poor treatment of acute COPD exacerbations is substantial, affecting quality of life and leading to work impairment and reduced productivity. The exacerbation rate among COPD patients receiving outpatient treatment was approximately 50%, while the hospitalization rate ranged between 8.78 and 35.60%. Addressing high smoking rates and air pollution is essential for improving disease prevention and management efforts to alleviate the burden associated with COPD (18, 19).

Research has examined various outcomes in patients with poor treatment acute exacerbations of chronic obstructive pulmonary disease. Some of these include poor outcomes such as symptom worsening, increased mortality, and complications for hospitalized COPD patients (15). Other noted outcomes include the deterioration of physical health, poor prognosis, and extended hospital stays (20).

Although a single study in Ethiopia addressed mortality and its associated factors related to acute exacerbations of chronic respiratory diseases, it involved a small sample size. Therefore, this study aims to assess the poor treatment outcomes of acute exacerbations of chronic obstructive pulmonary disease and their associated factors among admitted patients.

## Methods

### Study area and period

The study was conducted at the Debre Markos Comprehensive Specialized Hospital (DMCSH) from 7 April 2023 to 7 May. Located in East Gojjam Zone, Amhara Regional State, Northwest Ethiopia, in Debre Markos town, DMCSH is situated 299 km from Addis Ababa, the capital of Ethiopia, and 265 km from Bahirdar, the capital city of Amhara regional state. As one of the referral hospitals in Amhara Regional State, DMCSH potentially serves more than five million people and has 488 clinical staff and 183 administrative staff.

#### Study design

An institutional-based cross-sectional study design was employed.

#### Source population

The source population consisted of all patients diagnosed with acute exacerbation of COPD at DMCSH from 1 February 2018 to 30 January 2023.

#### Study population

All admitted patients diagnosed with exacerbation of chronic obstructive pulmonary disease at DMCSH during the same period were included in the study.

#### Inclusion criteria

All admitted patients who had a diagnosis of exacerbation of COPD in DMCSH during the 5-year period from 1 February 2018 to 30 January 2023 were included in the study.

#### Exclusion criteria

Patients younger than 18 years, those referred to other facilities, those who left the hospital against medical advice, and those with incomplete or lost records regarding major variables (death or survival) were excluded from the study.

#### Sample size determination

The sample size for the study was calculated using a single population proportion formula. A 95% confidence level and a 5% margin of error were considered, with 50% used as the estimated proportion due to the lack of similar studies. The calculation is as follows:


$$\mathrm{Therefore}:n=\frac{\left(\frac{Z\alpha }{2}\right)2\;P\left(1-P\right)}{d2}=\left(1.96\right)2\;\frac{0.5\left(0.5\right)}{\left(0.05\right)2}=384$$

where *n* = sample size, P = 50%, d = marginal error between sample and population, which is 0.05, and Z *α*/2 = critical value at 95% confidence interval, which is 1.96.

### Sampling techniques and procedures

**Figure 1:**
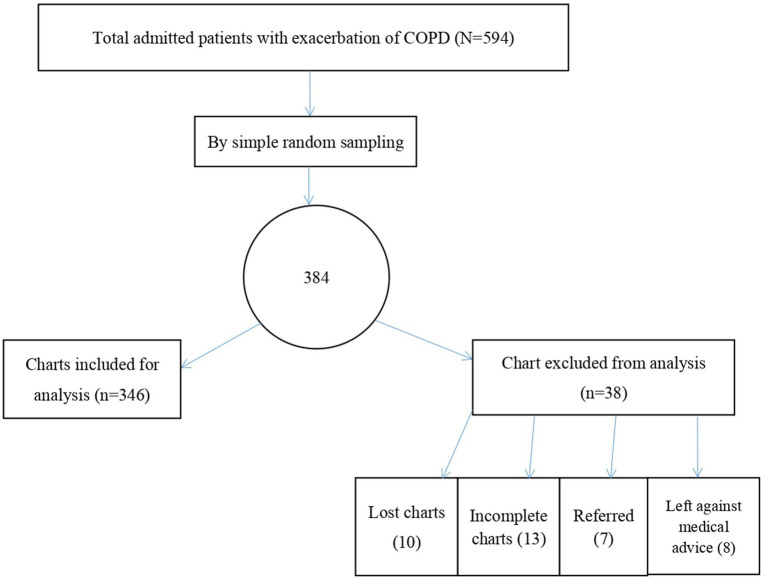
Sampling technique to assess the treatment outcome of acute exacerbation of COPD and its associated factors among admitted patients at the Debre Markos comprehensive specialized hospital, 2023.

A simple random sampling technique was utilized to select study participants. During the 5-year period starting on 1 February 2018, a total of 594 patients with acute exacerbations of COPD were admitted to the medical ward of DMCSH. Study participants were selected using computer-generated random numbers, with medical registration numbers serving as the sampling frame (Figure 1).

### Dependent variable

The dependent variable was the treatment outcome for acute exacerbations of COPD, categorized as either “good” or “poor”.

### Independent variables

The independent variables included **sociodemographic factors** (age, sex, residence, and occupation), **behavioral-related factors** (cigarette smoking), and **clinical and treatment-related factors**. These factors consisted of comorbidities (HIV/AIDS, heart disease, diabetes mellitus, hypertension, and chronic kidney disease), respiratory symptoms (cough, wheezing, shortness of breath, and cyanosis), vital signs of the patient, oxygen saturation, duration of illness, length of hospital stay, medications (cephalosporin [ceftriaxone], macrolide [azithromycin/clarithromycin], long and short-acting beta 2 agonists, corticosteroids), and investigation modalities (chest x-ray, ECG, ECHO, WBC count, hematocrit, serum creatinine, and platelet count).

### Operational definitions

#### Poor outcome

It is defined as the development of at least one complication (e.g., respiratory failure, pulmonary edema, cor pulmonale, and polycythemia) and/or death during hospitalization (21, 22).

#### Good outcome

This is defined as patients being discharged alive without any complication (e.g., respiratory failure, pulmonary edema, cor pulmonale, and polycythemia) (21, 22).

#### Oxygen saturation

The target range for oxygen saturation in COPD patients is between 88 and 92. Low oxygen saturation is defined as <88%, while normal saturation is ≥88% (23).

#### Length of hospital stay

Length of stay is categorized as normal (≤7 days) or prolonged (>7 days) for patients with acute exacerbation of COPD (24).

#### Blood pressure

Normal blood pressure is defined as between 90–120/60–80 mmHg, hypertensive as ≥130/80 mmHg, and hypotensive as <90/60 mmHg (25).

#### Age

Patients were classified into age groups of 18–34, 35–64, and ≥ 65 years (26).

### Data collection procedure

Four BSc nurses from DMCSH were selected to collect data, and one MSc nurse was appointed to supervise the data collectors daily. Patient records from patients admitted to the medical ward were retrieved from the medical ward logbook during the study period. The data collectors accessed the patients’ medical records from the hospital card room to fill out a structured questionnaire.

### Data collection tools

Data were collected from patient charts using a structured checklist. The English version of the checklist was adapted from various previous studies (21, 22). The checklist included sociodemographic characteristics, behavioral-related factors, and clinical- and treatment-related factors. Before the data collection process, the tool was organized based on the content validity index (CVI) format and sent to two internists by e-mail for evaluation. Based on their responses, item content validity index (I-CVI) scores were calculated by dividing the expert agreement by the number of experts. Finally, the average I-CVI scores of all items were calculated. Following this procedure, the content validity index was determined to be 0.83, indicating that the tool was acceptable. Moreover, reliability was assessed using Cronbach’s alpha, which yielded a value of 0.95.

### Data quality assurance

To ensure the quality of the data, the following measures were taken. Before the data were collected, the checklist was pretested on 5% of a sample size of patient charts at the Debre Markos Comprehensive Specialized Hospital, leading to modifications and edits to the checklist. The four data collectors and one supervisor received 1 day of training regarding the objectives and contents of the checklist. During the data collection process, both the supervisor and the principal investigator verified the completeness of the data daily.

### Data processing and analysis

The collected data were coded, entered into EPI Data version 4.6, and exported to Statistical Product and Service Solution (SPSS) version 26 for analysis. Descriptive statistics were used to summarize the data in terms of frequencies, percentages, medians, and interquartile ranges. Binary logistic regression analysis was conducted to identify associations between the dependent and independent variables. Variables with a *p*-value of <0.25 in the bivariable logistic regression analysis were selected as candidates for multivariable logistic regression, while those variables with a *p*-value of <0.05 were considered statistically significant. The adjusted odds ratio (AOR) with 95% confidence intervals (CI) was used. The Hosmer-Lemeshow test for logistic regression was conducted to examine the goodness of fit of the model, with the data fitting the model (x2 = 9.975, df = 8, *p*-value = 0.267). Multicollinearity was assessed using variance inflation factors, with all variables having a VIF of <10. The results are presented in the form of text, tables, and charts.

## Results

### Sociodemographic characteristics

In this study, a total of 346 out of 384 participants were included, resulting in a response rate of 90.1%. Of the participants, 217 (62.7%) were men. The median age of the study participants was 55 years, with an interquartile range (IQR) of (45–65) years. More than half of the participants, 185 participants (53.5%) were rural residents, and nearly one-third, 106 participants (30.6%), were farmers (Table 1).

**Table 1:** Sociodemographic characteristics of participants who were admitted to the Debre Markos comprehensive specialized hospital with the diagnosis of acute exacerbation of chronic obstructive pulmonary disease, 2023 (*N* = 346).

Variable	Categories	Frequency (100%)
Age	18–34	93 (26.9)
	35–64	153 (44.2)
	65 and above	100 (28.9)
Sex	Male	217 (62.7)
	Female	129 (37.3)
Residence	Urban	161 (46.5)
	Rural	185 (53.5)
Occupation	Farmer	106 (30.6)
	Housewife	82 (23.7)
	Government employee	90 (26.0)
	Merchant	51 (14.7)
	Other^a^	17 (4.9)

a Industrial worker, daily worker.

### Presenting signs and symptoms, durations of illness, and comorbid risk factors

The median duration of illness was 10 years, with an IQR of 8 to 15 years, while the median length of hospital stay was 9 days, with an IQR of 6 to 13 days. Cough was the most common presenting symptom, reported by 341 patients (98.6%), followed by wheezing in 282 (81.5%) and breathlessness in 255 patients (73.7%). During the study period, a total of 142 (41%) patients had a comorbidity, with approximately half of these patients, 69 (48.6%), suffering from hypertension.

On admission, 81 patients (23.4%) presented with elevated blood pressure. Additionally, 131 (37.9%) patients had a pulse rate exceeding 100 beats per minute, 333 patients (96.2%) had a respiratory rate of over 20 breaths per minute, and 105 patients (30.3%) had a temperature above 37.5 degrees Celsius. At discharge, 140 (40.5%) respondents had an oxygen saturation below 88% without supplemental oxygen (Table 2).

**Table 2:** Presenting signs and symptoms and comorbid factors of patients who were admitted to the Debre Markos comprehensive specialized hospital with the diagnosis of acute exacerbation of chronic obstructive pulmonary disease, 2023 (*N* = 346).

Variable	Category	Frequency (100%)
Comorbidity	Yes	142 (41)
	No	204 (59)
Type of comorbidity	Hypertension	69 (48.6)
	Congestive heart failure	33 (23.2)
	Diabetes mellitus	24 (16.9)
	AIDS^a^	11 (7.8)
	Other	5 (3.5)
Cough	Yes	341 (98.6)
	No	5 (1.4)
Sputum production	Yes	223 (64.5)
	No	123 (35.5)
Breathlessness	Yes	255 (73.7)
	No	91 (26.3)
Chest tightness	Yes	174 (50.3)
	No	172 (49.7)
Wheezing	Yes	282 (81.5%)
	No	64 (18.5)
Crepitation	Yes	249 (72)
	No	97 (28)
Cyanosis	Yes	129 (37.3)
	No	217 (62.7)
Oxygen saturation	Low (<88%)	140 (40.5)
	Normal (≥88%)	206 (59.5)
Length of hospital stay	≤7 days	187 (54)
	>7 days	159 (46)
Blood pressure	Normal	260 (75.1)
	Hypertensive	81 (23.4)
	Hypotensive	5 (1.4)
Pulse rate	Normal (60–100 bpm)	208 (60.1)
	Tachycardia (>100 bpm)	131 (37.9)
	Bradycardia (<60 bpm)	7 (2)
Respiratory rate	Normal (12–20brpm)	13 (3.8)
	Tachypnea (>20brpm)	333 (96.2)
Temperature	Normal (36.5–37.5^0^c)	229 (66.2)
	Hyperthermia (>37.5^0^c)	105 (30.3)
	Hypothermia (<36.5^0^c)	12 (3.5)

a HIV/AIDS: human immunodeficiency/acquired immunodeficiency syndrome.

### Investigation results of patients admitted with AECOPD

Of the patients, 115 (33.2%) exhibited an increased white blood cell count, 55 (15.9%) had a low hematocrit level, and 45 (13%) had an elevated serum creatinine level (Table 3).

**Table 3:** Laboratory investigation of patients who were admitted to the Debre Markos comprehensive specialized hospital with the diagnosis of acute exacerbation of chronic obstructive pulmonary disease, 2023 (*N* = 346).

Variable	Category	Frequency (100%)
WBC Count	Normal (5–11 × 10^3^/ul)	149 (43.1)
	Leukocytosis (>11,000)	115 (33.2)
	Leukopenia (<5,000)	82 (23.7)
Hematocrit level	Normal (36–52)	250 (72.3)
	High (>52)	41 (11.8)
	Low (<36)	55 (15.9)
Platelet count	Normal (150–450 × 10^3^/ul)	261 (75.4)
	High (>450 × 10^3^/ul)	29 (8.4)
	Low (<150 × 10^3^/ul)	56 (16.2)
Serum Cr level	Normal (0.5–1.3 mg/dL)	236 (68.2)
	High (>1.3 mg/dL)	45 (13)
	Low (<0.5 mg/dL)	65 (18.8)

**Figure 2:**
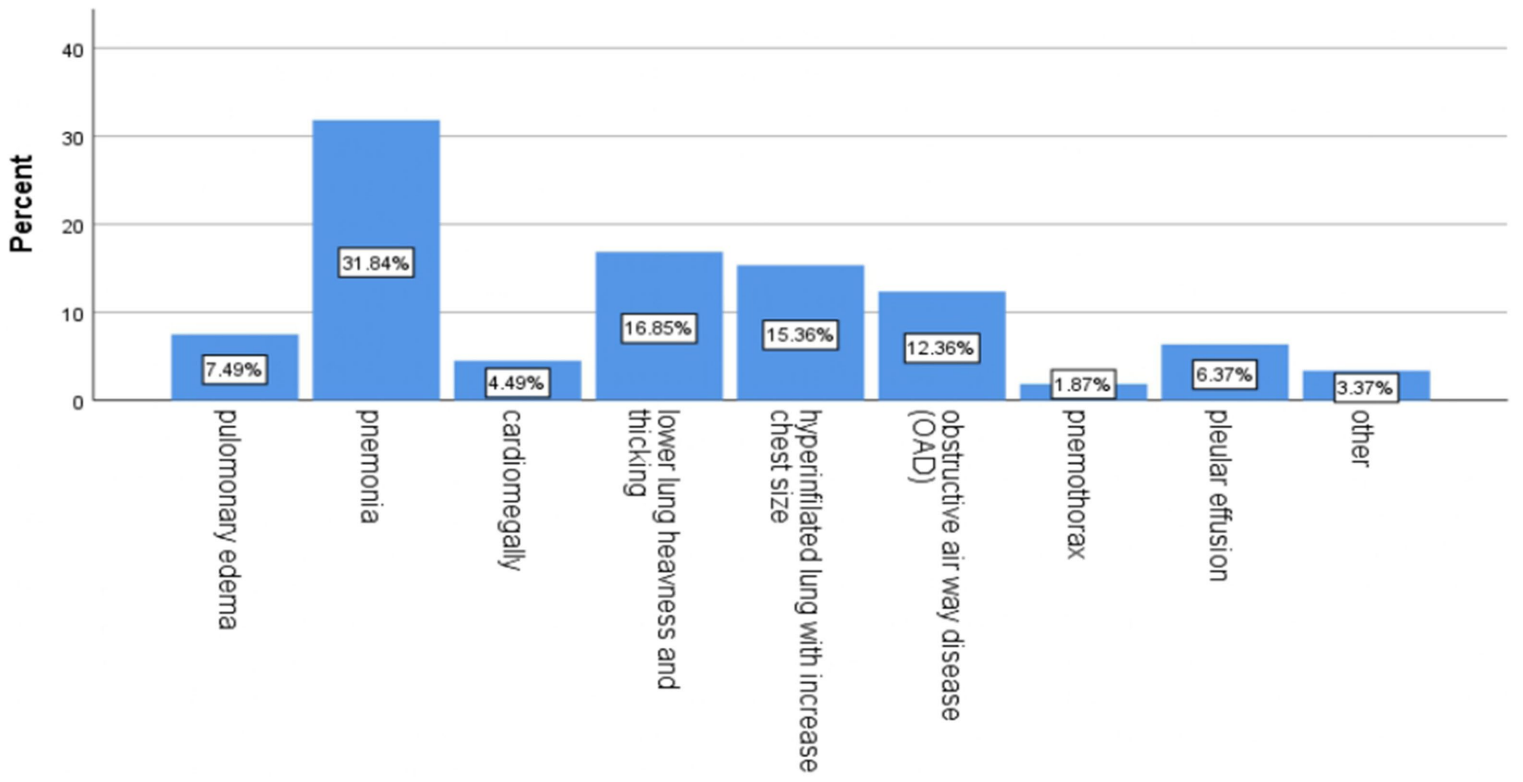
Proportion of chest x-ray findings among patients who were admitted to the Debre Markos comprehensive specialized hospital with a diagnosis of acute exacerbation of chronic obstructive pulmonary disease, 2023. * Other: lung fibrosis, lower lung opacity, and infiltration.

**Figure 3:**
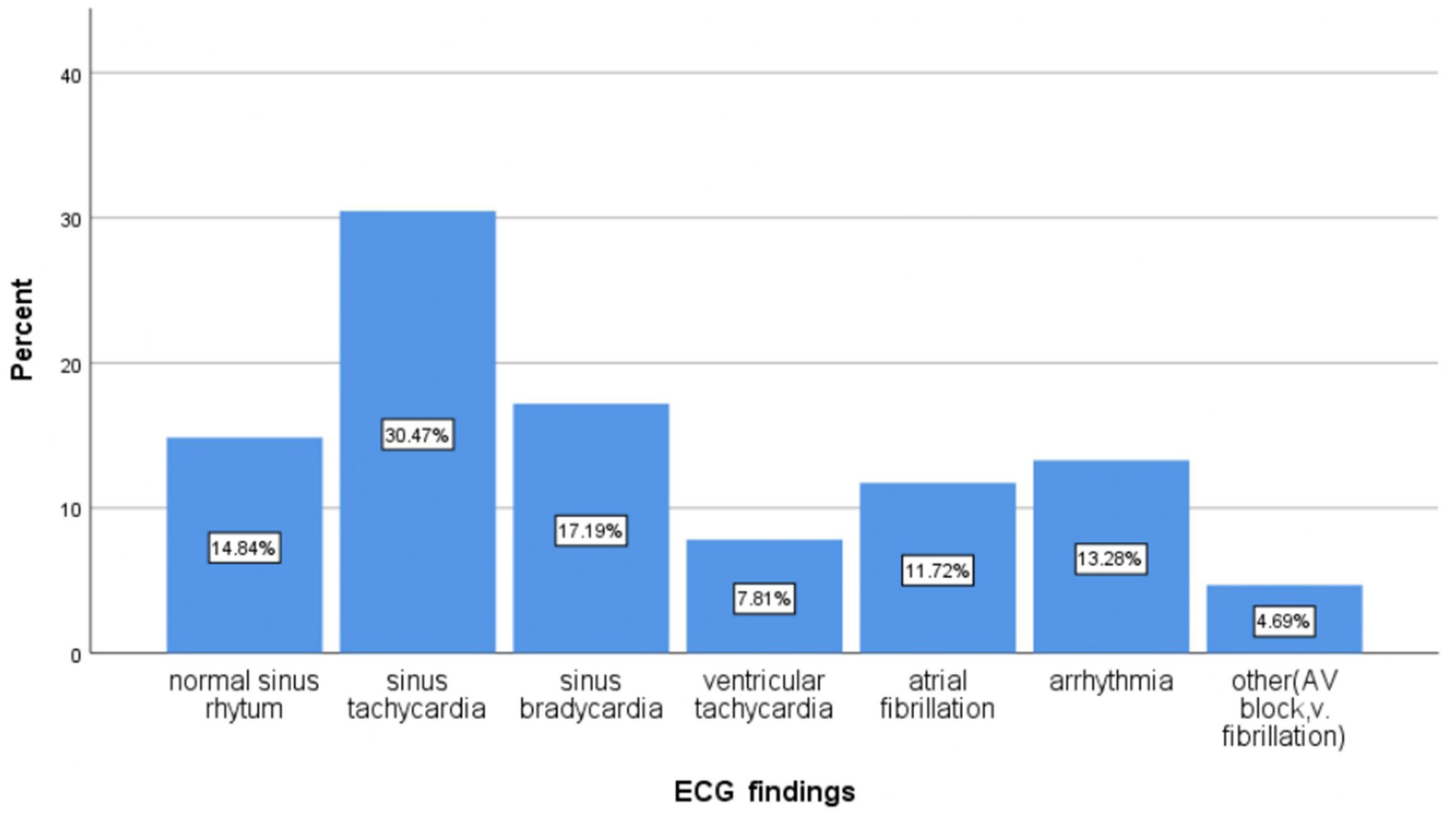
Proportion of electrocardiogram findings in patients who were admitted to the Debre Markos comprehensive specialized hospital with a diagnosis of acute exacerbation of chronic obstructive pulmonary disease, 2023.

**Figure 4:**
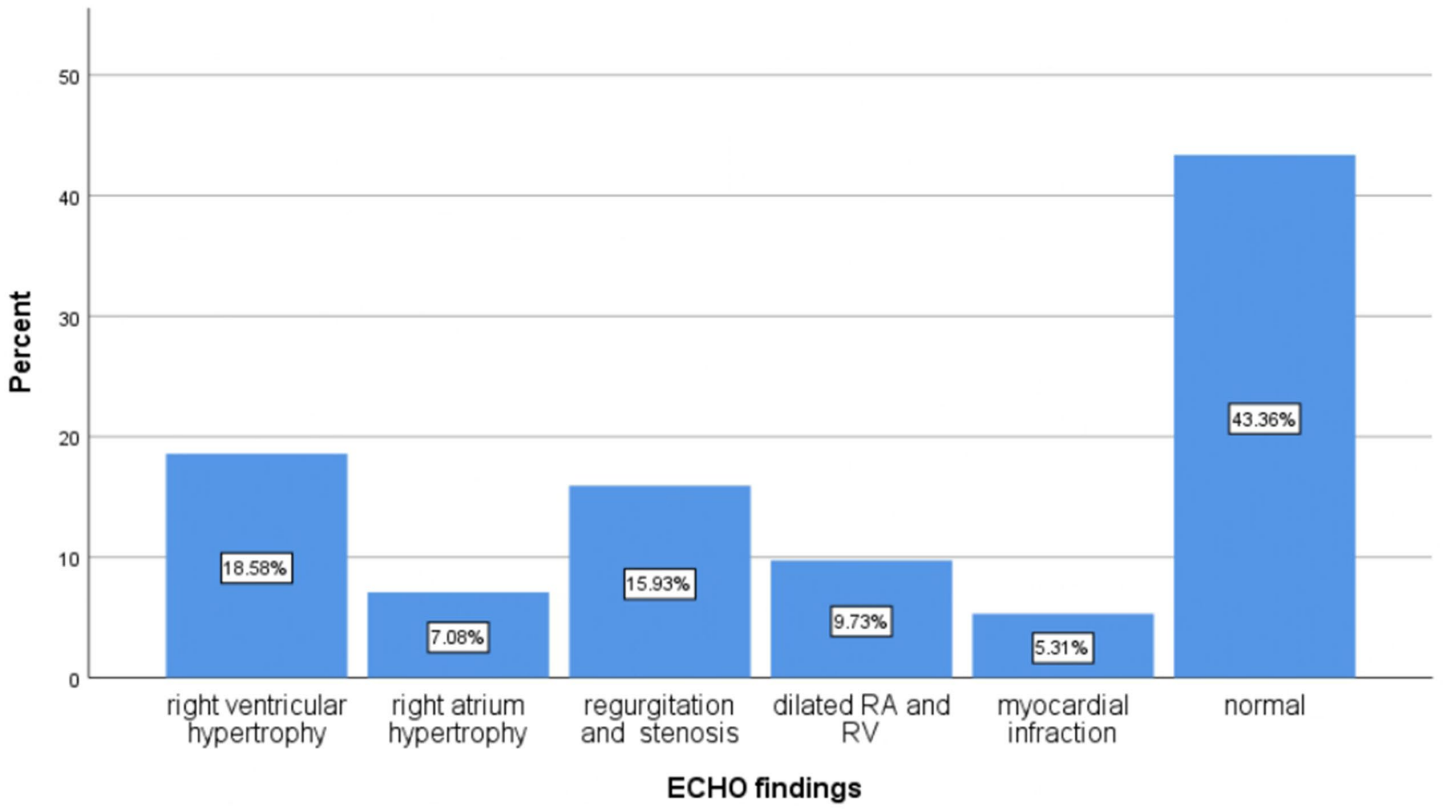
Proportion of echocardiography findings of the patients who were admitted to the Debre Markos comprehensive specialized hospital with diagnosis of acute exacerbation of chronic obstructive pulmonary disease, 2023.

Chest x-rays (CXRs) were performed on 267 patients (77.2%), ECG on 128 patients (37%), and ECHO on 113 patients (32.7%) (Figures 2–4 shows common findings from CXR, ECG, and ECHO, respectively).

### Treatment-related factors

All 346 patients received oxygen therapy. Among the respondents, 296 patients (85.5%) were treated with antibiotics, with 257 patients (86.8%) receiving a combination of cephalosporin (ceftriaxone) and macrolide (azithromycin/clarithromycin). All 346 patients were also treated with bronchodilators, and more than half, specifically 187 (54%), received long-acting beta-2 agonists in conjunction with corticosteroids (Table 4).

**Table 4:** Commonly prescribed drugs for patients who were admitted to the Debre Markos comprehensive specialized hospital with the diagnosis of acute exacerbation of chronic obstructive pulmonary disease, 2023 (*N* = 346).

Variable	Category	Frequency (100%)
Antibiotic	Cephalosporin (ceftriaxone) with macrolide (azithromycin/clarithromycin)	257 (86.8)
	Augmentin with macrolide	21 (7.1)
	Vancomycin	18 (6.1)
Bronchodilator	LABA (salmeterol/formaterol puff) + corticosteroid^a^	187 (54)
	SABA (salbutamol puff)^b^ + corticosteroid	146 (42.2)
	Other (long and short) acting muscarinic antagonists	13 (3.8)

a LABA (long-acting beta_2_ agonist), corticosteroid (hydrocortisone, beclomethasone, and prednisolone tablet).

b SABA (short-acting beta_2_ agonist).

### Outcomes in hospitalized patients with acute exacerbations of COPD

Of the 346 admitted patients with acute exacerbations of chronic obstructive pulmonary disease, 99 patients developed poor outcomes. Consequently, the proportion of poor outcomes due to exacerbations of chronic obstructive pulmonary disease at the Debre Markos Comprehensive Specialized Hospital was 28.6% (95% CI, 23.9–33.3). Of these patients, 74 (21.4%) developed complications, which included respiratory failure, pulmonary edema, cor pulmonale, and polycythemia. Additionally, 88 (25.4%) of the 346 patients admitted with acute exacerbations of COPD died (Table 5).

**Table 5:** Poor treatment outcomes of acute exacerbation of chronic obstructive pulmonary disease in patients at the Debre Markos comprehensive specialized hospital, 2023 (*N* = 346).

Variable	Category	Frequency (100%)
Complication	Yes	74 (21.4)
	No	272 (78.6)
Type of complication	Respiratory failure	25 (33.8)
	Pulmonary edema	19 (25.7)
	Cor pulmonale	18 (24.3)
	Polycythemia	5 (6.8)
	Other^a^	7 (9.5)
Condition of patient at discharge	Recovered	258 (74.6)
	Died	88 (25.4)
Treatment Outcome	Poor	99 (28.6)
	Good	247 (71.4)

a Myocardial infraction, pneumothorax.

### Factors associated with poor treatment outcomes

The following variables were identified in the bivariable analysis as being associated with poor outcomes in patients experiencing exacerbations of COPD, with a significant *p*-value of <0.25: age, sex, residence, occupation, wheezing, blood pressure at admission, white blood cell count, oxygen saturation, duration of illness, comorbidities, and length of the hospital stay.

Multivariable logistic regression analysis was performed on all independent variables associated with the bivariable analysis to determine their impact on poor outcomes in exacerbating COPD patients while controlling for other variables. The results showed that age 65 years and older (AOR = 3.9; 95% CI: 1.577–9.712), low oxygen saturation (AOR = 9.0; 95% CI: 4.438–18.341), a length of hospital stay >7 days (AOR = 3.9; 95% CI: 1.977–7.709), and the presence of comorbidities (AOR = 2.6; 95% CI: 1.287–5.209) were significantly associated with poor treatment outcomes, with a *p*-value of <0.05 (Table 6).

**Table 6:** Bivariable and multivariable logistic regression analyses for associated factors of poor treatment outcomes of acute exacerbation of chronic obstructive pulmonary disease at the Debre Markos comprehensive specialized hospital, 2023 (*N* = 346).

Variables	Category	Outcome		COR (95% CI)	AOR (95% CI)	*p*-value
		Poor	Good			
Age of the patient in years	18–34	21 (22.6%)	72 (77.4%)	1	1	
	35–64	24 (15.7%)	129 (84.3%)	0.6 (0.33–1.22)	0.7 (0.28–1.69)	0.428
	65 & above	54 (54%)	46 (46%)	4 (2.15–7.52)	3.9 (1.57–9.71)	**0.003**
Length of hospital stay	≤7 days	22 (11.8%)	165 (88.2%)	1	1	
	>7 days	77 (48.4%)	82 (51.6%)	7 (4.09–12.11)	3.9 (1.97–7.70)	**0.001**
Presence of comorbidities	Yes	58 (40.8%)	84 (59.2%)	2.7 (1.70–4.43)	2.6 (1.28–5.20)	**0.008**
	No	41 (20.1%)	163 (79.9%)	1	1	
Respondent’s oxygen saturation	Low (<88%)	75 (53.6%)	65 (46.4%)	8.8 (5.10–15.01)	9 (4.43–18.34)	**0.001**
	Normal (≥88%)	24 (11.7%)	182 (88.3%)	1	1	

Bold values are significant factors.

## Discussion

The main purpose of the current study was to assess poor treatment outcomes and associated factors of acute exacerbation of chronic obstructive pulmonary disease at the Debre Markos Comprehensive Specialized Hospital. According to this study, the overall proportion of poor treatment outcomes for acute exacerbation of COPD at the hospital was 28.6% (95% CI, 23.9–33.3).

This rate is higher than that found in studies conducted at Jacksonville University of North Florida Hospital (21%) (27), France (7.4%) (28), the Netherlands (8%) (29), India (12%) (5), Japan (21.3%) (30) and Southern-Eastern Nigeria (10.8%) (6). The possible explanation for this discrepancy may lie in differences in the level of healthcare settings, treatment approaches, and quality of care. The aforementioned countries have better quality of care and use pulmonary function tests (spirometry) more extensively than this study. Spirometry can guide therapy for COPD, enable monitoring of disease progression, and facilitate appropriate treatment (31).

Conversely, this study’s findings are lower than those from Spanish hospitals 36.8% (21), Geneva (39%) (32), Malaysia (59.5%) (33), two Nepalese hospitals (36.7%) (34), Southern Thailand (37.3%) (35) and Western Australia (56.8%) (36). The variation may be attributed to the sample size; the number of study participants in this research was greater than that in the studies conducted in Malaysia, Nepal, Southern Thailand, and Western Australia. In contrast, the studies in Geneva and Nepal were multicenter studies. The selection of study participants from emergency and intensive care units may also explain the poorer outcomes observed. Additionally, the ability of healthcare facilities to diagnose or detect complications may have contributed to the differences in outcomes compared to this study.

Patients aged 65 years and above were found to be 3.9 times (95% CI, 1.577–9.712) more likely to have poor outcomes compared to those aged 18–34 years. This finding is supported by studies conducted in France (37), Spain (38), the Netherlands (29), Japan (3), Malaysia (33), Nepal (34), and Jimma (22). The higher incidence of comorbidities in older individuals and age-related degenerative changes in lung function—such as reductions in respiratory muscle strength, vital capacity, and the total alveolar surface—may accelerate pulmonary function deterioration in older patients with COPD. Additionally, a declining immune response may contribute to worse disease progression (39, 40).

According to this study, patients with comorbidities were 2.6 times (95% CI, 1.287–5.209) more likely to develop poor outcomes than those without comorbidities. This finding is supported by studies conducted in the United States (41), India (16), the north and south regions of Kyrgyzstan (42), and Nigeria (6). A possible explanation is that comorbidities significantly impact clinical course, complications, and outcomes, leading to increased drug-related toxicities. Furthermore, comorbidities can increase the frequency of hospitalization and healthcare costs, resulting in socioeconomic deprivation and inadequate social support, ultimately affecting patient outcomes (43–45). Effective management of these comorbidities can help reduce the poor outcomes associated with the disease.

The current study showed that patients who stayed in the hospital for longer than 7 days were 3.9 times (95% CI, 1.977–7.709) more likely to develop poor outcomes than those who stayed for less than 7 days. This finding is supported by studies from the United States (41), Turkey (46) and studies conducted by Cheng et al. (47).

The possible reason for this may be the presence of comorbidities, which indirectly prolong hospital stays, preventing patients from being discharged sooner, increasing the risk of healthcare-associated infection and complications, and raising hospital costs. This situation negatively affects patients’ economic conditions and social support systems. Consequently, these factors may hinder patients’ ability to afford hospital costs and receive adequate and quality care (48–50).

Therefore, reducing the length of hospital stays is a key strategy for improving healthcare utilization and achieving better patient outcomes. This contrasts with findings from studies conducted in Nepal (34), Japan (3), and Jimma (51), which found no significant association between the length of hospital stay and the outcome of COPD exacerbations.

This study found that patients with low oxygen saturation were 9 times (95% CI, 4.438–18.341) more likely to develop poorer outcomes than those with normal oxygen saturation. This finding is supported by studies from Turkey (46), Wellington Hospital in Florida (52) and Sohag University Hospital in Egypt (53). The possible explanation may be that COPD patients with low oxygen saturation develop hypoxemia and hypoxia, which impair the functioning of various body organs and increase the risk of cognitive impairment, loss of consciousness, and even death. Consequently, these patients may have worse outcomes (54).

## Strengths and limitations of the study

The study utilized data recorded by healthcare professionals. Consequently, some key variables, such as the patient’s condition at discharge (alive or deceased), were incomplete. Additionally, it was challenging to measure other variables, such as exposure to cigarette smoke, khat chewing, and alcohol consumption since the data were collected through chart reviews, and these variables were not documented in the charts.

## Conclusion and recommendations

Approximately one-third of patients treated for acute exacerbations of COPD at the Debre Markos Comprehensive Specialized Hospital experienced poor treatment outcomes. Statistically significant associations were found between poor outcomes and factors such as age (65 years and older), comorbidities, hospital stay longer than 7 days, and low oxygen saturation levels.

To enhance treatment outcomes for COPD patients, healthcare professionals should prioritize managing comorbidities, ensuring adequate oxygen supply to improve saturation, and providing specialized care for older patients. Additionally, further research using prospective study designs is necessary to address the limitations of the secondary data utilized in this retrospective study, which may hinder the generalizability of the findings to the broader population.

## Data Availability

The data supporting the findings of this study are available upon request from the corresponding author and will be shared in accordance with the journal’s data sharing policies.
